# The Effects of Qigong for Hypertension: A Meta-Analysis of Randomized Controlled Trials

**DOI:** 10.1155/2021/5622631

**Published:** 2021-10-08

**Authors:** Xiaosheng Dong, Zhenguo Shi, Meng Ding, Xiangren Yi

**Affiliations:** ^1^Department of Sport and Health, School of Physical Education, Shandong University, Jinan 250061, China; ^2^College of Physical Education, Shandong Normal University, Jinan 250014, China

## Abstract

**Background:**

Hypertension has been a global public health problem. Qigong as a complementary and alternative therapy is often used to reduce blood pressure. The aim of this meta-analysis was to investigate the effects of Qigong on blood pressure in hypertensive patients.

**Methods:**

Six electronic resource databases were searched from inception to January 2019, and randomized controlled trials of Qigong on hypertension were retrieved. Meta-analysis was conducted according to the guidelines of the Cochrane Collaboration, and Review Manager 5.3 was applied. Two researchers independently identified articles to include based on inclusion/exclusion criteria, data extraction, and quality evaluation.

**Results:**

Fourteen studies, with 829 individuals, were included. The meta-analysis demonstrates that, compared with no exercise, Qigong has significant positive effects on systolic blood pressure (mean difference = −8.90, 95% CI (−12.13, −5.67), *P* < 0.00001) and diastolic blood pressure (mean difference = −5.02, 95% CI (−7.88, −2.17), *P* < 0.00001). There is, however, no significant difference between Qigong and other aerobic exercises in reducing blood pressure.

**Conclusion:**

Qigong can effectively reduce blood pressure levels. Longer-term engagement in the practice has an even better effect in hypertension patients. However, the conclusion of this study still needs to be verified by more high-quality studies.

## 1. Introduction 

Hypertension is a global public health problem and an essential preventable factor of cardiovascular diseases [1, 2]. According to relevant studies, nearly 1.13 billion people worldwide were affected by high blood pressure in 2015 [3]. Moreover, the total medical cost for rehabilitation of hypertension and its complications worldwide is currently about 370 billion US dollars per year, which causes a substantial personal, family, and socio-economic burden [4]. Previous studies have shown that the probability of patients with hypertension being able to keep their blood pressure within the normal range with drug therapy alone is still very low [5]. Therefore, to control blood pressure, many patients have to take multiple antihypertensive drugs concurrently. However, this practice increases the financial burden and may have unforeseen side effects [6].

Modern medical research suggests that appropriate physical activity can effectively control and prevent the occurrence of hypertension and its complications [7]. Studies have shown that physically active people have a lower blood pressure than sedentary people [8]. Besides, the Eighth Joint National Committee issued guidelines on hypertension which pointed out that regular exercise can effectively control blood pressure, prevent, and treat hypertension [5]. Hence, exercise therapy plays an important role in the treatment of hypertension and blood pressure control.

Qigong is a traditional fitness method that originated from ancient China [9]. It has the elements of body movement, mind guidance, and breath control, and the combination of these elements can achieve the function of physical and mental adjustment [10]. Furthermore, it has the effect of lowering blood pressure, controlling complications, and reducing mortality [11–13]. Three [12–14] published studies systematically evaluated and meta-analyzed the effect of Qigong on hypertension, but these studies have some inadequacies. Therefore, it is necessary to include more high-quality studies to further clarify the effect of Qigong on hypertension. Besides, although two studies [15, 16] have discussed whether exercise has a dose-response relationship with the risk of hypertension, whether exercise, including Qigong, had a dose-response relationship with blood pressure level of hypertension patients was not studied. Hence, this study basically explores the dose-response relations between quantity of motion in Qigong exercise amount (minutes) and blood pressure level of hypertension patients by meta-analysis.

## 2. Methods

### 2.1. Aims

The aim of this meta-analysis was to investigate the effects of Qigong on hypertension and explore the dose-response relations between quantity of motion in Qigong exercise amount and blood pressure level of hypertension patients using meta-analysis.

### 2.2. Design

This study mainly contains a meta-analysis of all randomized controlled trials involving Qigong to treat hypertension published in English or Chinese. The review is carried out under the instructions of the Cochrane Collaboration [17].

### 2.3. Search Methods

This study searched the Cochrane Library, China Science and Technology Journal Database (CQVIP), MEDLINE via PubMed, Web of Science, Wan Fang Data, and China National Knowledge Infrastructure (CNKI), to find randomized controlled trials exploring Qigong's effects on hypertension, and retrieved data to January 2019. Besides, references to studies are also considered to prevent omission. There are two kinds of search terms, namely, target (hypertension) search term and intervention (Qigong) search term, which were slightly adjusted according to the specific database. A combination of subject search and free search was used in all retrieval. Seven presearches are used to facilitate search strategies. Certain search terms include Qigong, Baduanjin, Wuqinxi, high blood pressure, hypertension, blood pressure, systolic blood pressure (SBP), and diastolic blood pressure (DBP).

#### 2.3.1. Inclusion Criteria

(1) Studies in Chinese and English. (2) The randomized controlled trial (journal articles, conference articles, and dissertations) shall be included. (3) With no restrictions on gender or age of subjects with hypertension. (4) Blood pressure (systolic and diastolic) was measured. (5) Qigong was tested as the main intervention compared to any other control. (6) The major intervention in the control group must be one of no exercise (maintenance of original lifestyle) or other aerobic exercises (aerobic exercise except traditional sports including Tai chi and Qigong) or resistance exercises. (7) Patients without serious hypertension-related complications.

#### 2.3.2. Exclusion Criteria

(1) Nonrandomized controlled trials. (2) Studies that did not include data of indicators (systolic blood pressure and diastolic blood pressure) to be discussed in this study. (3) The major intervention in the control group was not one of no exercise (maintenance of original lifestyle) or other aerobic exercises (aerobic exercise except traditional sports including Tai chi and Qigong) or resistance exercises. (4) The major intervention in the experimental group was not Qigong. (5) Patients with serious hypertension-related complications.

### 2.4. Search Outcome

**Figure 1:**
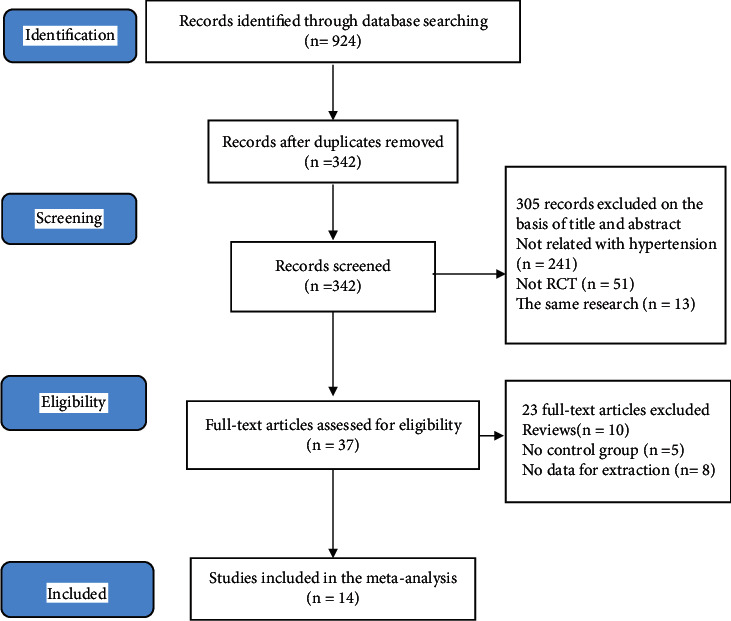
Flow diagram of study selection and identification.

Databases such as The Cochrane Library, VIP, MEDLINE via PubMed, Web of Science, Wan Fang Data, and CNKI were retrieved according to the inclusion and exclusion criteria and the effects of fitness Qigong on blood pressure. 924 potentially relevant documents were found in the searched database, and 342 documents were retained after the duplication of the literature was removed. Among these, 305 were deleted by reading titles and abstracts, and 23 were excluded after reading the full text according to the inclusion criteria. Finally, a total of 14 trials were included [18–31], including 5 English trials [27–31] and 9 Chinese trials [18–26]. The study selection process is shown in Figure 1.

### 2.5. Data Extraction

During the process, two researchers did independently and if disagreements arose, the third person was involved to analyze and decide whether to include and exclude. Data extracted include information of the first author, publication date, past published journals, paper title, intervention measures of experimental group and control group, intervention period, number of experimental group and control group, and info of patients (age and gender). Hypertension indicators shall include systolic and diastolic blood pressure.

### 2.6. Quality Appraisal

During the data extraction process, the quality of the included studies was evaluated using the Cochrane Collaboration's tool for assessing risk of bias. Evidence from studies was classified as having a low, unclear, and high risk of bias. This process was conducted independently by two researchers on quality assessment and resolves differences by soliciting the opinions of a third person.

**Figure 2:**
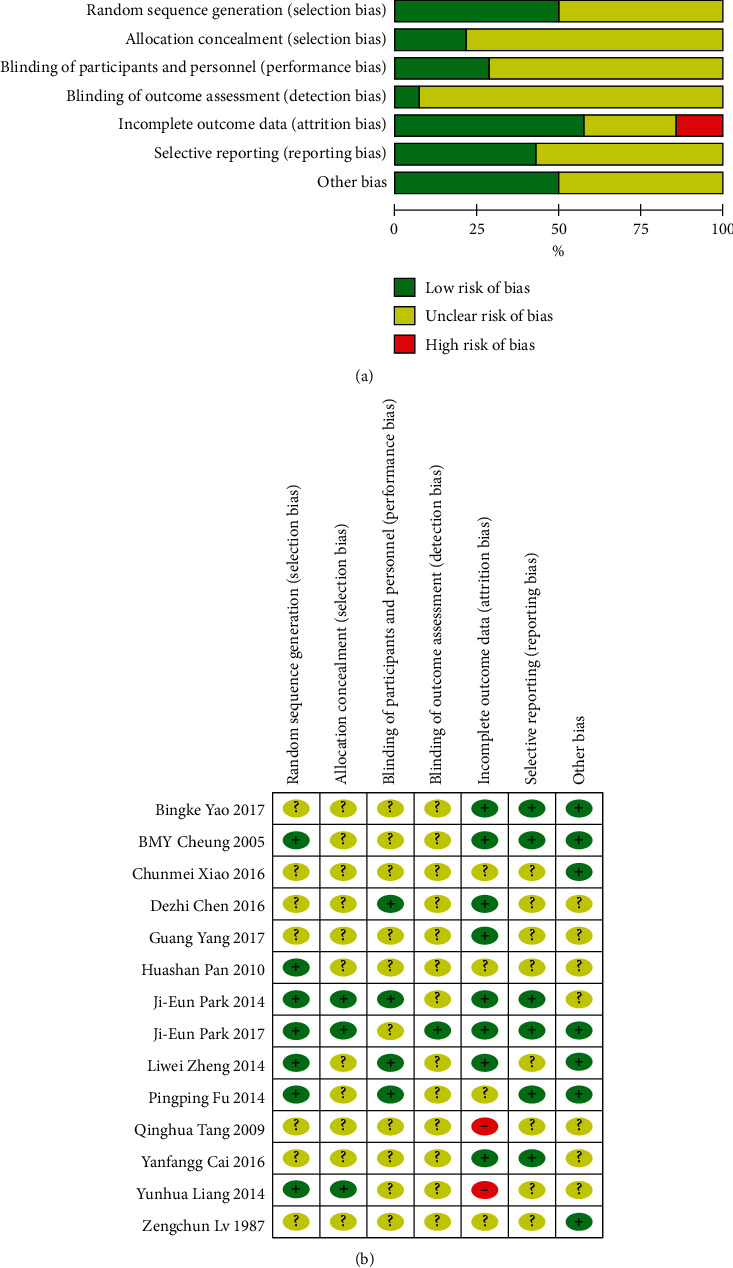
Assessment of risk of bias. (a) Risk of bias graph and (b) risk of bias summary.

Outcomes of each included study are shown in Figure 2. In terms of “random sequence generation,” there were 7 studies classified as low risk, and 7 studies were regarded as an unclear risk. With respect to “allocation concealment” and “blinding of participants and personnel,” only 3 studies were classified as low risk, and the remaining were classified as unclear risk. In terms of “incomplete outcome data,” 8 studies were rated low risk, 4 were rated unclear risk, and another 2 were rated high risk. In terms of “selective reporting,” 6 studies were rated low risk, and 8 were rated unclear risk. In terms of “other biases,” 7 studies were rated low risk, and 7 were rated unclear risk.

### 2.7. Statistical Analysis

Review Manager 5.3 (Nordic Cochran Centre, Copenhagen, Denmark) and Stata 14.0 (Stata Corp., College Station, TX) software were used for meta-analysis. The heterogeneity test between the results of included studies was *χ*^2^. Statistical homogeneity between the results of each study indicated that when *P* > 0.1 and *I*^2^ <50%, the fixed effect model was adopted. When heterogeneity showed *P* > 0.1, *I*^2^ >50%, heterogeneity was analyzed by subgroup analysis. If there was no heterogeneity between subgroups, a fixed effect model was used. When statistical heterogeneity existed among subgroups in the included studies, a random effect model was used for meta-analysis. If there was too much heterogeneity between groups, descriptive analysis was performed. Sensitivity analysis was applied if necessary. Possible publication bias was assessed using funnel plot and Egger's test. The funnel plots were constructed based on the logarithm of the effect sizes against the standard error of the effect sizes for studies included in the meta-analyses. The trim and fill method was applied to correct the effect of possible bias. Mean differences (MD) for each study were calculated by subtracting the score difference in the control group (systolic blood pressure and diastolic blood pressure) from the score difference in the intervention group. Negative values were indicative of improvements (reductions) in systolic blood pressure or diastolic blood pressure. In addition, 95% confidence intervals (CI) were calculated.

In order to reveal a dose-response relationship between Qigong and blood pressure lowering among patients with hypertension, this study compared the amount of exercise of people doing Qigong and that of inactive people and conducted stratified subgroup analysis. Because Qigong can be considered as a kind of moderate-intensity aerobic exercise [31], in this study, we did not take into account the differences in exercise intensity of all selected studies, and the total exercise amount is indicated by total exercise minutes. The included study was separated into five subgroups based on the total exercise amount, namely, the very small exercise group is for exercising less than 1,500 minutes; the small total minutes exercise group is for exercising between 1,501 and 3,000 minutes; the medium total minutes exercise group is for exercising between 3,001 and 4,500 minutes and the large total minutes exercise group is for exercising between 4,501 and 6,000 minutes; the superlarge total minutes exercise group is for exercising more than 6,001 minutes. The total exercise amount (minutes) = Qigong exercise time (minutes) per day ^∗^ Qigong exercise days per week ^∗^ total of Qigong exercise weeks (calculated as 4 weeks per month).

## 3. Results

### 3.1. Characteristics of the Included Studies

A total of 14 randomized controlled trials, with a total of 829 subjects, were included. The ages of these subjects were within 40.0–71.0 years, with a minimum follow-up time of 10 weeks and a maximum follow-up time of 1 year. All patients in intervention groups maintained routine medication and received Qigong intervention at the same time. 5 articles have adopted the Baduanjin [18, 21–23, 27]; 1 article adopted the Yijinjing [19], 1 article adopted the Liuzijue [20], and 7 articles did not report the type of Qigong [24–26, 28–31]. The frequencies and times of Qigong exercise in all included articles have been reported. The control groups were separated into nonexercise groups and other aerobic exercise groups. The subjects in other aerobic exercise groups did walking and jogging as an intervention [21, 24, 29] (see Table 1).

### 3.2. Outcomes

#### 3.2.1. Systolic Blood Pressure

**Figure 3:**
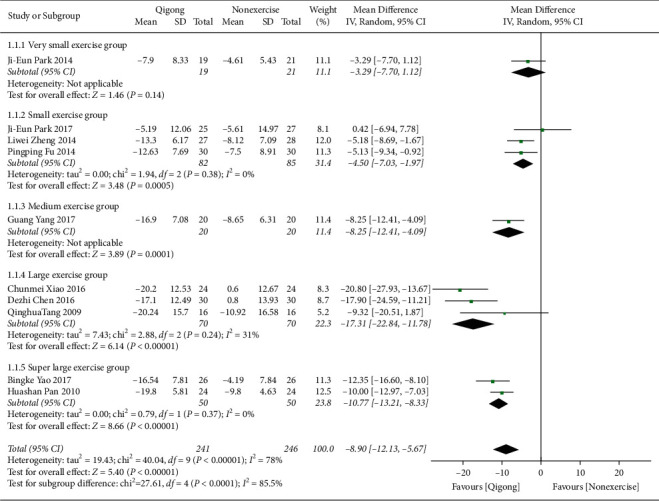
The meta-analysis for comparing SBP between Qigong and nonexercise. CI: confidence interval; SD: standard deviation.

*(1) Qigong versus Nonexercise*.  Figure 3 shows that data obtained from 11 [18–20, 22, 23, 25–28, 30, 31] related studies (*n* = 600) could be combined, but the data heterogeneity was relatively high. According to the subgroup analysis of the total amount of exercise, the heterogeneity of the superlarge exercise group was as high as 88%. By sensitivity analysis of the data, it can be inferred that the data of Yanfang Cai 2016 was the main source of heterogeneity in the superlarge exercise group. Accordingly, we excluded data from the studies and reported the data. Finally, data were collected from 10 studies (*n* = 487), and the results showed that systolic blood pressure in the Qigong group was lower than that in the nonexercise group, with statistically significant difference (MD = −8.90, 95% CI (−12.13, −5.67), *P* < 0.00001). With an exercise amount of 1–6000 minutes, the subgroup pooled effect size increased with the increase of total exercise amount. With exercise amount of 4501–6000 minutes, the subgroup pooled maximum effect size (MD = −17.31, 95% CI (−22.84, −11.78), *P* < 0.00001).

**Figure 4:**
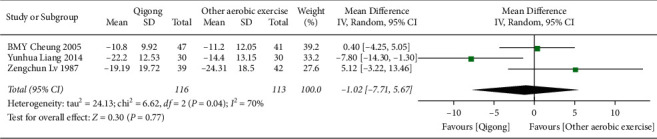
The meta-analysis for comparing SBP between Qigong and other aerobic exercises. CI: confidence interval; SD: standard deviation.

*(2) Qigong versus Other Aerobic Exercises*. As shown in Figure 4, three related studies (*n* = 229) [21, 24, 29] adopted the random-effects model for data pool, and the results showed no statistical difference between Qigong and other aerobic exercises in SBP (MD = −1.02, 95% CI (−7.71, 5.67), *P*=0.77).

#### 3.2.2. Diastolic Blood Pressure

**Figure 5:**
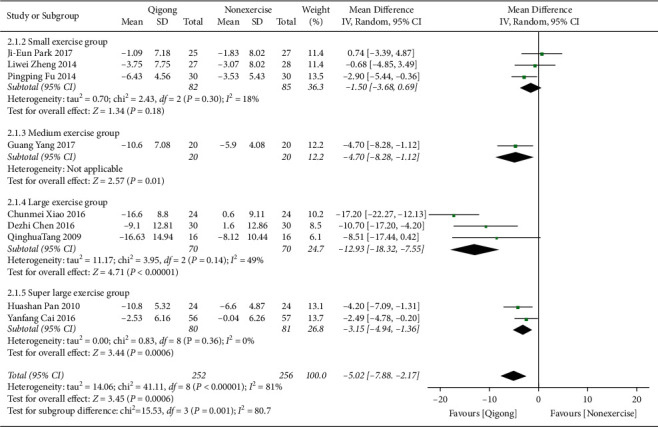
The meta-analysis for comparing DBP between Qigong and nonexercise. CI: confidence interval; SD: standard deviation.

*(1) Qigong versus Nonexercise*. Initially, the data of 10 studies [18–20, 22, 23, 25–28, 31] were pooled, but the data heterogeneity was relatively high. Through sensitivity analysis, it was speculated that the data of Bingke Yao 2017 was the main source of heterogeneity in a superlarge exercise group. Accordingly, the data of this study were excluded, and the data pool was processed again. Finally, the data of 9 studies (*n* = 508) were collected, and the results showed that DSP in the Qigong group was lower than that in the control group, and the difference was statistically significant (MD = −5.02, 95% CI (−7.88, −2.17), *P* < 0.00001). Above 3000-minute exercise amount, the subgroup pooled showed statistically significant difference (3,001–4,500 minutes: MD = −4.70, 95% CI [−8.28, −2.17], *P*=0.01; 4,501–6,000 minutes: MD = −12.93, 95% CI [−18.32, −7.55], *P* < 0.00001; more than 6,001 minutes: MD = −3.15, 95% CI [−4.94, −1.36], *P*=0.00006). With an exercise amount of 4501–6000 minutes, the subgroup pooled maximum effect size (MD = −12.93, 95% CI (−18.32, −7.55), *P* < 0.00001) (Figure 5).

**Figure 6:**
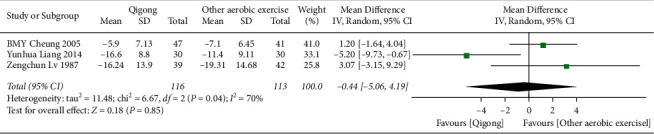
The meta-analysis for comparing DBP between Qigong and other aerobic exercises. CI: confidence interval; SD: standard deviation.

*(2) Qigong versus Other Aerobic Exercises*. Data from 3 [21, 24, 29] related studies (*n* = 229) could be synthesized as shown in Figure 6. The random-effect model was used for data combination. The results showed that Qigong had no better effect on DBP reduction than other aerobic exercises, and the difference was not statistically significant (MD = −0.44, 95% CI (−5.06, 4.19), *P*=0.85).

### 3.3. Publication Bias Evaluation

#### 3.3.1. Publication Bias Evaluation between Qigong and Nonexercise

**Figure 7:**
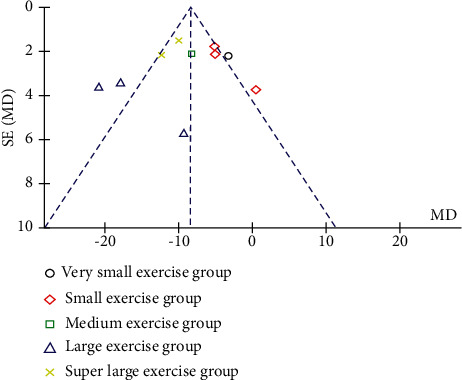
The systolic blood pressure in the Qigong and nonexercise of the funnel plots.

A funnel plot was used to analyze systolic blood pressure in the Qigong group and inactivity group. A total of 10 studies involving 487 subjects were included. These results indicate that the included studies were symmetrically distributed on both sides of the funnel plot (Figure 7). Analysis of the complete data set did not show any sign of publication bias (Egger's test *p*=0.147). A trim and fill analysis showed that no study was missed or trimmed. Overall, there was no evidence of publication bias in this analysis.

#### 3.3.2. Publication Bias Evaluation between Qigong and Other Aerobic Exercises

**Figure 8:**
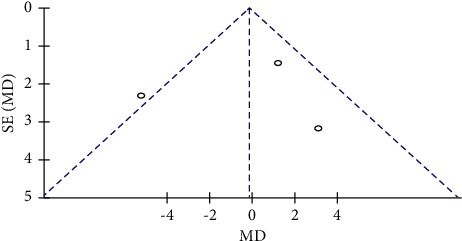
The diastolic blood pressure in the Qigong and other aerobic exercises of the funnel plots.

The diastolic blood pressure (DBP) was analyzed with funnel plots including 3 studies and 229 subjects. The results showed that the funnel plot included in the study was asymmetrically distributed on both sides, indicating a potential publication bias in the comparison of Qigong and other aerobic exercises (Figure 8).

## 4. Discussion

According to the results of this study, Qigong has a positive effect on the reduction of diastolic and systolic blood pressure indicators compared with no exercise in hypertension patients. This result is basically consistent with the results of three published studies on systematic evaluation and meta-analysis [12–14]. As a traditional exercise, Qigong can control and reduce the levels of blood pressure in hypertension patients.

From the perspective of modern sports medicine, based on the form of Qigong exercise and its exercise intensity, we conclude that it is one kind of regular and moderate intensity of aerobic exercises [10]. In this study, compared with no exercise, Qigong has obvious advantages in adjusting systolic and diastolic blood pressure. Therefore, Qigong can reduce blood pressure in hypertension patients. In addition, although the results of this study show that Qigong has no advantage in reducing systolic or diastolic blood pressure compared with other aerobic exercises, previous relevant studies have confirmed that aerobic exercise has a lowering effect on blood pressure [32]. Previous studies have shown that Qigong can regulate the levels of endothelial-derived factors (nitric oxide and endothelin-1), and the changes of systolic and diastolic blood pressure are related to the changes of nitric oxide and endothelin-1 [33]. Therefore, the effect of Qigong on the levels of nitric oxide and endothelin-1 may be a potential mechanism to reduce hypertension. Despite the lack of research to confirm the safety of Qigong, it is worth noting that none of the literature included in this study reported any adverse effects or side effects caused by the exercise of Qigong. As a physical and mental exercise originating from ancient times and with oriental cultural style, Qigong has some advantages compared with other aerobic exercises. For example, Qigong movements are simple and easy to learn, with moderate intensity and fewer requirements on the site and equipment. It can be practiced individually or collectively.

At present, there are few pieces of research at home and abroad on whether there is a dose-effect relationship between exercise (including Qigong) and blood pressure level in hypertension patients. However, a study showed a linear dose-response relationship between blood pressure levels and exercise levels, with higher exercise levels associated with lower blood pressure levels [34]. According to the results of this study, within a certain range, a higher total physical activity amount corresponds to a higher effect value with changes in the blood pressure level. Therefore, we believe that there is a dose-response relationship between total Qigong exercise amount and the reduction of a blood pressure level in hypertension patients, but the conclusion of this study needs to be verified by more high-quality studies. Moreover, the current studies show that the relationship between exercise intensity and blood pressure level is inconsistent. Some studies have shown that low-intensity exercise can reduce the systolic blood pressure level better [35]. Conversely, some studies have found that high-intensity interval training is equal to or even better than medium-intensity continuous training in reducing blood pressure [36, 37]. However, this study did not make a subgroup analysis of the exercise intensity of Qigong mainly because the included studies basically did not report the exercise intensity of Qigong and Qigong is a kind of moderate-intensity aerobic exercise, so it is necessary to explore the relationship between exercise intensity and blood pressure in future studies.

According to the meta-analysis results, the exercise amount of Qigong of 4501–6000 minutes has a better effect on the reduction of diastolic blood pressure and systolic blood pressure. Therefore, in order to better regulate the diastolic blood pressure and systolic blood pressure level, it is recommended to do 90–120 minutes of Qigong exercise every week for more than one year or 180–240 minutes of Qigong exercise every week for more than half a year. This recommended amount of exercise for Qigong is basically the same as 150 minutes of weekly aerobic exercise with moderate-intensity recommended in most guidelines of clinical practice [38].

This study found that Qigong exercise is positive and effective in reducing hypertension. In patients with hypertension, Qigong exercise for 90–120 minutes per week for more than one year or 180–240 minutes per week for more than half a year is the better treatment for hypertension. It must be pointed out, however, that these are general recommendations. It is worth noting that Qigong is easier to learn and has fewer side effects than other treatment methods for hypertension (standard exercise and drugs) [39]. Especially for elderly hypertensive patients with drug side effects, Qigong exercise is an effective exercise substitutive therapy.

Limitations of this study shall also be taken into account. Firstly, limited by objective conditions, only MEDLINE via PubMed, The Cochrane Library, Web of Science, CNKI, Wan Fang Data, and VIP were applied, so potential omissions may occur; secondly, the quality of included papers was low and the random distribution method and blind method was not described; thirdly, our study did not use metaregression to adjust for confounding factors; fourth, due to the large heterogeneity of the included studies, only random effect model can be adopted, which will have a certain impact on the results; fifth, some confidence intervals (CI) that are too wide may not estimate precise.

## 5. Conclusion

Qigong can effectively reduce blood pressure levels, and 90–120 minutes every week for more than one year or 180–240 minutes every week for more than half a year has a better effect in hypertension patients. Moreover, according to the subgroup analysis of the total Qigong exercise amount, there may be a dose-response relationship between Qigong exercise amount and blood pressure level in hypertension patients. However, the conclusion of this study still needs to be verified by more high-quality studies.

## Figures and Tables

**Table 1:** Characteristics and quality assessments of the included trials.

First author, year	Patients (total, E/C)	Age (mean (SD), E/C)	Experimental intervention	Control	Duration, sessions with supervision per week, time per session
Yanfang Cai, 2016	113, 56/57	60.89(9.71)/61.33(8.24)	Baduanjin	N	1 year, 5, 30–40 min
Bingke Yao, 2017	52, 26/26	45.89(6.36)/45.23(5.76)	Yijinjing	N	3 months, 7, 90 min
Guang Yang, 2017	40, 20/20	58.10(6.95)/57.70(6.30)	Liuzijue	N	12 weeks, 4, 90 min
Yunhua Liang, 2014	60, 30/30	54.8(7.6)/55.7(8.8)	Baduanjin	Walking	6 months, 10, 20 min
Liwei Zheng, 2014	55, 27/28	69.23(3.72)/70.06(3.51)	Baduanjin	N	12 weeks, 5, 30 min
Huashan Pan, 2010	48, 24/24	62.1(5.8)/61.4 (7.1)	Baduanjin	N	24 weeks, 10, 45 min
Zengchun Lv, 1987	81, 39/42	56.31/55.52	Qigong	Jogging	3 months, 7, 60 min
Pingping Fu, 2014	60, 30/30	57.93(6.63)/59.53(7.46)	Qigong	N	3 months, 6, 40 min
Qinghua Tang, 2009	24, 16/16	62.79(7.43)/63.84(8.12)	Qigong	N	6 months, 3–5, 30–60 min
Chunmei Xiao, 2016	48, 24/24	65.6 (7.8)	Baduanjin	N	6 months, 5, 40 min
Dezhi Chen, 2016	60, 30/30	65.4(17.2)/64.8 (15.3)	Qigong	N	6 months, 5, 44 min
BMY Cheung, 2005	88, 47/41	57.2 (9.5)/51.2(7.4)	Qigong	Walking	16 weeks, 7, 75 min
Ji-Eun Park, 2014	40, 19/21	52/54	Qigong	N	8 weeks, 3, 30 min
Ji-Eun Park, 2017	52, 25/27	54.52(6.96)/52.93(8.45)	Qigong	N	12 weeks, 5, 50 min

E: experimental group; C: control group; N: nonexercise; NR: not reported; min: minute; SD: standard deviation.

## Data Availability

Specific study data are available from the authors upon request.
